# Connexin36 Expression in the Mammalian Retina: A Multiple-Species Comparison

**DOI:** 10.3389/fncel.2017.00065

**Published:** 2017-03-09

**Authors:** Tamás Kovács-Öller, Gábor Debertin, Márton Balogh, Alma Ganczer, József Orbán, Miklós Nyitrai, Lajos Balogh, Orsolya Kántor, Béla Völgyi

**Affiliations:** ^1^Department of Experimental Zoology and Neurobiology, University of PécsPécs, Hungary; ^2^János Szentágothai Research CenterPécs, Hungary; ^3^Retinal Electrical Synapses Research Group, Hungarian Academy of Sciences (MTA-PTE NAP B)Pécs, Hungary; ^4^Department of Biophysics, University of PécsPécs, Hungary; ^5^High-Field Terahertz Research Group, Hungarian Academy of Sciences (MTA-PTE)Pécs, Hungary; ^6^Nuclear-Mitochondrial Interactions Research Group, Hungarian Academy of Sciences (MTA-PTE)Pécs, Hungary; ^7^National Research Institute for Radiobiology and RadiohygieneBudapest, Hungary; ^8^Department of Anatomy, Histology and Embryology, Semmelweis UniversityBudapest, Hungary; ^9^Department of Neuroanatomy, Institute for Anatomy and Cell Biology, Faculty of Medicine, University of FreiburgFreiburg, Germany; ^10^Department of Ophthalmology, New York University Langone Medical Center, New YorkNY, USA

**Keywords:** electrical synapses, gap junction, connexin36

## Abstract

Much knowledge about interconnection of human retinal neurons is inferred from results on animal models. Likewise, there is a lack of information on human retinal electrical synapses/gap junctions (GJ). Connexin36 (Cx36) forms GJs in both the inner and outer plexiform layers (IPL and OPL) in most species including humans. However, a comparison of Cx36 GJ distribution in retinas of humans and popular animal models has not been presented. To this end a multiple-species comparison was performed in retinas of 12 mammals including humans to survey the Cx36 distribution. Areas of retinal specializations were avoided (e.g., fovea, visual streak, area centralis), thus observed Cx36 distribution differences were not attributed to these species-specific architecture of central retinal areas. Cx36 was expressed in both synaptic layers in all examined retinas. Cx36 plaques displayed an inhomogenous IPL distribution favoring the ON sublamina, however, this feature was more pronounced in the human, swine and guinea pig while it was less obvious in the rabbit, squirrel monkey, and ferret retinas. In contrast to the relative conservative Cx36 distribution in the IPL, the labels in the OPL varied considerably among mammals. In general, OPL plaques were rare and rather small in rod dominant carnivores and rodents, whereas the human and the cone rich guinea pig retinas displayed robust Cx36 labels. This survey presented that the human retina displayed two characteristic features, a pronounced ON dominance of Cx36 plaques in the IPL and prevalent Cx36 plaque conglomerates in the OPL. While many species showed either of these features, only the guinea pig retina shared both. The observed similarities and subtle differences in Cx36 plaque distribution across mammals do not correspond to evolutionary distances but may reflect accomodation to lifestyles of examined species.

## Introduction

Electrical synapses have been discovered more than 50 years ago (Farquhar and Palade, 1965; Rosenbluth, 1965; Brightman and Reese, 1969; Raviola and Gilula, 1973). GJs are formed at close cell membrane appositions to allow the intercellular communication of neurons by the exchange of ions, second messengers and small molecules up to 1 kD (Furshpan and Potter, 1957; Watanabe, 1958). Six connexin (Cx) protein subunits establish connexon hemichannels and they pair up to form functional intercellular channels. The Cx constituents determine the unitary pore permeability, thus they ultimately determine the function that a particular GJ plays in the retinal circuitry (Söhl and Willecke, 2003; Bloomfield and Völgyi, 2009; Völgyi et al., 2013a). Over 20 mammalian Cx genes have been sequenced (Söhl and Willecke, 2003), that are named after the molecular weights between 21 and 70 kDa (Cx43, Cx26, etc.). In retinas of mammalian models, Cx36 and Cx45 have been found in both plexiform layers (Güldenagel et al., 2000; Petrasch-Parwez et al., 2004). They form GJs of both the primary and secondary rod pathways (Feigenspan et al., 2001, 2004; Güldenagel et al., 2001; Mills et al., 2001; Deans et al., 2002; Lee et al., 2003; Han and Massey, 2005; Lin et al., 2005; Maxeiner et al., 2005) and also ganglion-to-ganglion (GC-to-GC) and ganglion-to-amacrine cell (GC-to-AC) GJs (Hidaka et al., 2004; Schubert et al., 2005a,b; Völgyi et al., 2005, 2009, 2013a,b). In contrast, the high permeable Cx50 and Cx57 GJs connect horizontal cells to maintain an outer retinal syncytium (Hombach et al., 2004; Janssen-Bienhold et al., 2009; Dorgau et al., 2015; Bolte et al., 2016). While functions that GJs play in the retina seem conservative across vertebrate species (Völgyi et al., 2013a), the core of our knowledge stems from only a handful of popular species including the mouse, rat, rabbit, and monkey. Additionally, the great number of clinically relevant studies utilizing various model animals calls upon a comparison of the human retina with those of popular mammalian species. To gain a more general insight, a multiple-species comparison was performed using retinal tissues from 12 mammalian species (dog, ferret, cat, guinea pig, hamster, squirrel monkey, rat, human, rabbit, sheep, mouse, swine) to survey the distribution of the Cx36 protein subunit. In order to eliminate variations in Cx36 plaque distribution attributed to intraspecies regional location and retinal eccentricity, all samples were collected from the inferonasal retinal quadrants and retinal specializations (fovea, area centralis, visual streak) were avoided. In order to further standardize measurements, samples were taken from the centralmost region of inferonasal quadrants except for samples collected for experiments in which central and peripheral areas were compared in human and squirrel monkey retinas. Observed pan-retinal features included the expression of Cx36 in both synaptic layers and the inhomogenous distribution of Cx36 puncta in the IPL. The Cx36 labeling pattern in the OPL differed considerably among mammals. In general, Cx36 puncta in the OPL were rare and small in rod dominant carnivore and rodent retinas. In contrast, human and cone dominant guinea pig retinas displayed robust Cx36 labels in the OPL. The inhomogenous distribution of Cx36 plaques in the IPL and the robust plaque formation in the OPL were two pronounced features exhibited by the human retina. While several species showed either of these two features, only the guinea pig retina shared both. In addition, the overall Cx36 distribution is largely conservative across various mammalian species but there are subtle species-to-species differences in the density and the dominancy of the Cx36 plaque locations suggesting adaptations to the various lifestyles of the examined species. Again, the above observed differences in Cx36 plaque distribution were not attributed to variations in species-specific central retinal architecture (e.g., fovea, visual streak, area centralis) or retinal eccentricity of samples.

## Materials and Methods

### Animals and Sample Collection

The species used in the present study are (name; binominal nomenclature; strain; number; sex; age): mouse (*Mus musculus*, C57BL/6J, 10 male, 3 months), rat (*Rattus norvegicus*, Wistar, 5 male, 4–6 months), golden hamster (*Mesocricetus auratus*, 2 male, 3 months), guinea pig (*Cavia porcellus*, 5 male, 10 months), rabbit (*Oryctolagus cuniculus*, New Zealand white, 2 male, 1 female, 5 months), sheep (*Ovis aries*, Hungarian Merino, 2 male, 1 female, 6 months), cat (*Felis silvestris catus*, 2 male, 1 female, 10 months), dog (*Canis lupus* fam., Beagle, 2 male, 1 female, 14–20 months), ferret (*Mustela putorius furo*, 2 male, 1 female, 10 months), swine (*Sus scrofa domesticus*, 1 female, 6 months), squirrel monkey (*Saimiri sciureus*, 1 male, 1 female, 4 and 9 years). In order to rule out effects of circadian rhythmicity on the expression level of the Cx36 protein as well as Cx36 plaque patterns, all animal dissections were performed between 9 and 11 AM. All the animals were young (except squirrel monkey), mature (post-pubertant), sexually intact, clinically healthy animals – acting in other studies as non-treated, negative controls. Animals were first anesthetized by intravenous ketamine hydrochloride and xylazine (10 and 1 mg per kg of body weight, respectively), then euthanized with intravenous (dogs, cats, sheep) or intracardial (mice, rats, golden hamsters, ferrets, rabbits, guinea pigs) administration of T-61 ad us. vet. injection (T-61 is a registered veterinary drug exclusively utilized for euthanasia, Bayer Hungária Kft., Budapest, Hungary). The eyes and the retinas were removed and processed for either WB or IHC. Animal handling, housing, and experimental procedures were reviewed and approved by the ethical committee of the University of Pécs (BA02/2000-6/2006) and the Semmelweis University (22.1/1068/3/2010). All animals were treated in accordance with the ARVO Statement for the Use of Animals in Ophthalmic and Vision Research. All efforts were made to minimize pain and discomfort during the experiments. Human tissue from patients (*n* = 3, age: 27–59, postmortem time 2–3 h: without known eye disease) was collected immediately after the removal of corneas for transplantation in accordance with the tenets of Declaration of Helsinki. All personal identifiers were removed and samples were coded prior to histological processing. Experimental protocols with human eyes were approved by the ethics committee of the Semmelweis University (TUKEB 58/2014).

### Western Blot Sample Preparation

Retina samples were homogenized in 300 μl radioimmunoprecipitation assay (RIPA) buffer (10 mM phosphate buffer pH 7.2, 1% NP-40, 1% Na-deoxycholate, 0.1% SDS, 0.15 M NaCl, 2 mM EDTA, with freshly added components: 2 μg/mL aprotinin, 0.5 μg/mL leupeptin, 2 mM sodium vanadate, 20 mM sodium fluoride, 0.5 mM dithiothreitol, and 10 mM phenylmethylsulfonyl fluoride). Protein concentrations were determined with BCA reagent using a standard curve from bovine serum albumine. After dilution to 1 μM with RIPA-buffer, 10 μl of DTT (0.5 M) and LDS Sample Loading Buffer (4X) and heating to 70°C (10 min) proteins were separated with 10% SDS-PAGE (100/150 V) with a protein ladder. The PA gel was semi-dry blotted to PVDF membrane (120 mAh, 30 min) and blocked in TBS-Tween20 with 1% BSA and 5% non-fat milk powder for 45 min. Rabbit polyclonal to Cx36 (Abcam: ab86408, Cambridge, UK) antibody was used for the WB reaction.

### Histological Preparation and Immunohistochemistry

Immediately after sacrifice (9–11 AM), the superior pole of the eyes was marked and followed by quick enucleation. Eyeballs were cut at the ora serrata, lens and vitreous body was removed. For WBs, retina was detached from the posterior eyecup and frozen immediately. Samples were stored at -80°C. For histological analyses, eyecups were fixed with 4% paraformaldehyde for 2 h at +4°C. The eyecups were cut in halves or quadrants from humans and animals with large eyeballs (rabbit, sheep, cat, dog). Samples were taken from central locations of the inferonasal retinal quadrants for all species. Central specializations including visual streak, area centralis and fovea were avoided in order to obtain comparable datasets. In addition, peripheral areas were also sampled for human and squirrel monkey retinas to examine eccentricity related variations in Cx36 plaque distributions. Fixative was rinsed out with phosphate-buffered saline (PBS, pH 7.4), eyecups were soaked in 15%, then 30% sucrose in PBS at 4°C and embedded in Tissue Tek (Sakura Finetek Europe B. V., Alphen aan den Rijn, Netherlands). Tissue blocks were stored at -80°C for further processing. 12 μm sections were cut in the meridional plane on a cryostat (Leica CM 1860, Leica Microsysteme, Wetzlar, Germany), sections were mounted on gelatin coated slides and stored at -20°C until further processing. Defrosted sections were treated with the primary antibodies (mouse anti-Cx36, 1:1,000, MAB3045 – Merck Hungary, Budapest, Hungary; rabbit anti-CaR 1:1,000, AB-5054 Merck Hungary, Budapest, Hungary) for overnight at room temperature, washed thoroughly with PBS, treated with the appropriate secondary antibodies (goat-anti mouse IgG conjugated with Alexa488, goat anti-rabbit IgG conjugated with Alexa546; Life Technologies Hungary Ltd., Budapest, Hungary), washed and and mounted with Vectashield mounting medium (Vector Laboratories Inc., Burlingame, CA, USA).

### Sequence Analysis and Connexin Dendrogram

Cx36 amino acid sequences of examined species were derived from the NCBI Protein database, or transcribed from corresponding mRNA sequences. We also entered homologous Cx45 and Cx57 sequences for certain animals as negative controls. Amino acid sequences homologous with the 71–110 amino acid (aa) fragment of the white perch Cx36 protein were aligned using CLC Main Workbench^1^ (CLC bio, QIAGEN Company) software (parameters: gap open = 10; gap ext. = 1; end gap = 0). A subsequent Neighbor-joining analysis was performed with 1000x bootstrap. We performed a multiple alignment on the corresponding aa sequences, to obtain a mammalian consensus sequence to compare it with the white perch Cx36 aa sequence.

We prepared dendrograms showing sequence similarities of the Human Cx36 gene to: (i) Cx36 genes of other mammalian species; (ii) other Cx genes whose protein products also form GJs in the human retina (Cx43, Cx45, Cx59, Cx62) and (iii) the white perch (Morone americana) as its Cx35/36 protein sequence was utilized to generate the monoclonal anti-Cx36 antibody (the immunogen was Cx36 71-110 aa). Nucleotide sequences (Cx43 Human: NM_000165; Cx45 Human: NM_005497; Cx59 Human NM_030772; Cx62 Human NM_032602; Cx36 Human BC143788; Cx36 Mouse NM_010290, Cx36 Ferret XM_004796539; Cx36 Rat NM_019281; Cx36 Rabbit XM_002718001; Cx36 Dog XM_544602; Cx35 White perch AF059183; Cx36 Cat XM_003987299; Cx36 Guinea Pig XM_003475663; Cx36 Hamster XM_005087948) were derived from NCBI Nucleotide database and were aligned using CLC Main Workbench software (parameters: gap open cost = 20.0; gap extension cost = 2.0). A subsequent Neighbor-joining analysis was performed with 1000x bootstrap.

### Confocal Microscopy and Image Processing

Sections were inspected by using LSM 710 confocal laser scanning microscope using a PlanApochromat 63x objective (NA: 1.4) (Carl Zeiss Inc., Jena, Germany) with appropriate laser lines and filter settings. Final pictures were constructed with Adobe Photoshop 7.0 or with Adobe Illustrator CS3 (San Diego, CA, USA). Only minor adjustments with respect to brightness and contrast were made, which did not alter the appearance of the original material.

### Densitometry

Sections (10 μm thickness; central areas of inferonasal retinal quadrants) with perfect perpendicular sectioning axes were selected. Collapsed images were obtained by using a z-projection tool (with the maximum intensity function; ImageJ 1.47 software, NIH) in the selected z-stacks. For densitometric analyses representative areas (*n* = 5 for each optical section) were selected by using a rectangular (150 μm × 20 μm) selection tool. Most blood vessels, non-specific stainings, holes or impurity were avoided to minimize methodoligal artifacts. Five consecutive optical sections (each section is 0.5 μm thick; each selected area makes up a 2.5 μm ^∗^ 150 μm ^∗^ 20 μm = 7.5 ^∗^ 10^3^ μm^3^ volume) in each area were collapsed and densitometric measurements were performed by using the analyze/plot profile function of Image J. The mean Cx36 intensity values were normalized in order to detect IPL strata with the highest and lowest Cx36 densities for a cross-species comparison.

## Results

### Homology of Anti-Cx36 Recognition Sites across Vertebrates

**FIGURE 1:**
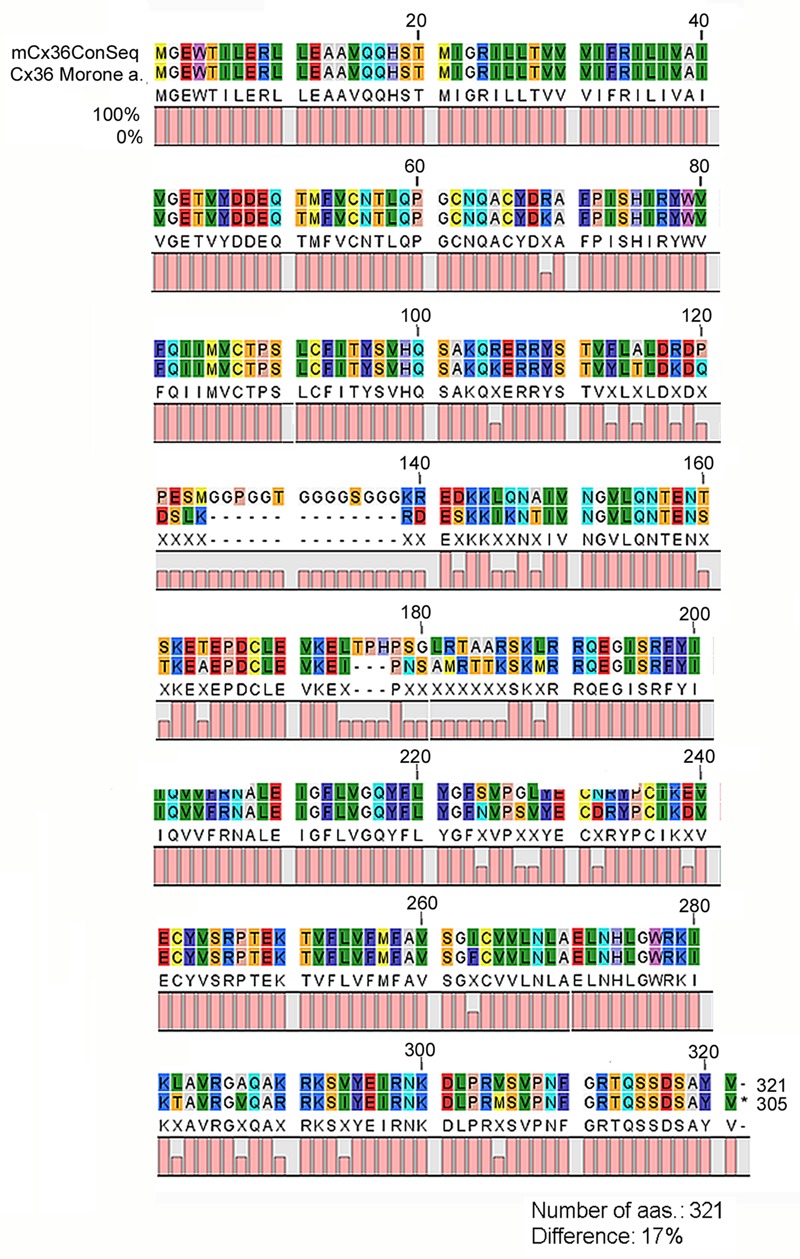
**Comparative amino acid sequence analysis of the mammalian consensus Cx36 and the Perch Cx36 protein.** The Cx36 Mammalian Consensus (mCx36ConSeq) represents the most prevalent aa sequence derived from the examined mammalian Cx36 sequences (NP_034420, NP_062154, XP_003987348, XP_003475711, XP_544602, XP_004751103, XP_001924792, XP_003935798, NP_065711, XP_005088005, XP_002718047, XP_004010475). When the white perch (Morone americana) Cx36 protein was compared to mCx36ConSeq a high similarity was observed with a ∼17% pairwise difference. Various aas appear with distinctive color codes in the figure. 0, 50, and 100% was calculated for each aa that was missing, altered (represented with an X symbol) and remained unchanged between the mCx36ConSeq and the perch protein, respectively. mCx36ConSeq, mammalian Cx36 consensus sequence; *Morone* a., *Morone americana*.

In this study, we performed IHC by using an anti-Cx36 primary antibody produced to recognize a 71–110 aa sequence of the perch Cx36 protein. Due to the conservative sequence of the target antigene the same antibody has widely been used to recognize Cx36 GJs in other vertebrates including mammals (Mills et al., 2001; Deans et al., 2002; Kovács-Öller et al., 2014). To validate the applicability of this serum to specifically detect the Cx36 sequence in mammalian species we first performed an analysis to compare the amino acid sequence of the immunoreactive region of the perch Cx36 protein to those of the examined mammalian species (see Materials and Methods). Rather than comparing each mammalian sequence to the perch protein a mammalian Cx36 consensus sequence (mCx36ConSeq) was created to represent the most prevalent Cx36 amino acid sequence of the examined mammals (**Figure 1**; see Materials and Methods). The alignment and subsequential analysis revealed that the 321 amino acid long mCx36ConSeq contained a poly-G area in the middle (between aa. 125 and 138), which is missing from the original immunogen perch protein. The mCx36ConSeq also had additional 39 amino acids that differed from the perch Cx36, most of which located in the second half of the mCx36ConSeq following the poly-G area. Altogether, the mCx36ConSeq showed 17% difference from the original perch protein, but there was only one amino acid difference in the 71–110 amino acid antibody interacting region (**Figure 1**). Thus, the antiserum binding region is rather conservative and likely does not alter the antigene-antiserum reaction.

**FIGURE 2:**
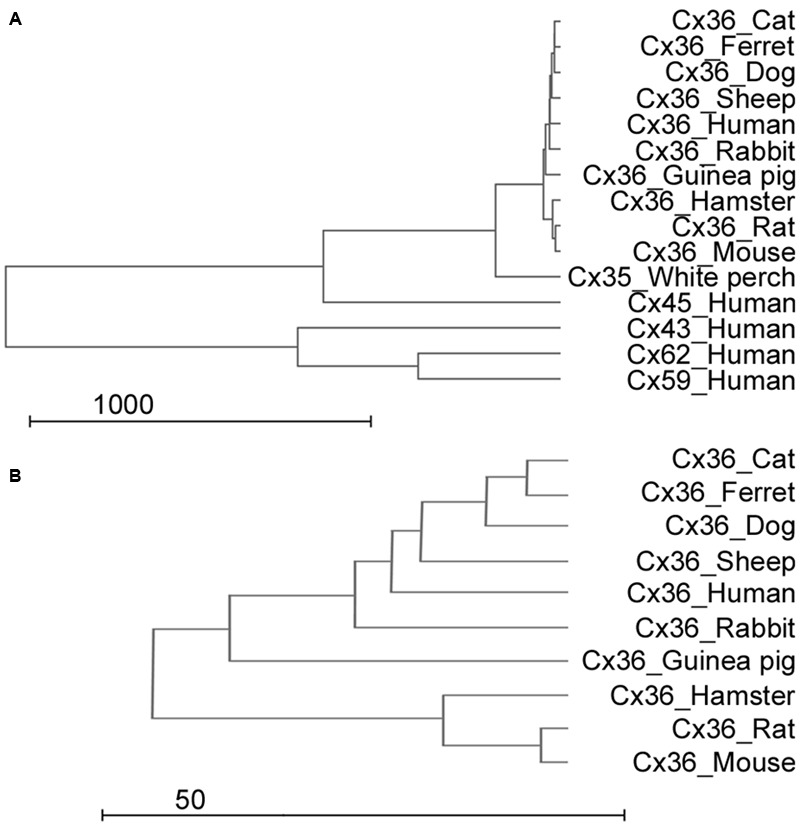
**Sequence analysis of Cx subunits. (A,B)** The dendrogram shows sequence similarities of the human Cx36 gene to other mammalian Cx36 genes, other human Cx genes (Cx43, Cx45, Cx59, and Cx62) and the white perch Cx36 sequence (with whole scale in **A** and focused to the Cx36 clade in **B**). Scale is arbitrary and represents relative genetical distance.

Results of the previous analysis indicated that the utilized antiserum should recognize Cx36 molecules in all examined species, however, cross-reaction with other expressed Cx molecules were not excluded. By using a bootstrap analysis a representative dendrogram was generated to visualize the differences in amino acide sequences of various Cx molecules (**Figure 2A**). The dendrogram clearly showed that differences occur mostly when Cx36 fragments were compared to Cx45 and Cx57 (orthologs) and less frequently when they were compared to Cx36 sequences of other species (paralogs). This was the case even for the perch Cx36 that lacked the above mentioned poly-G sequence (see above). The analysis thus predicted a very low likelihood for the monoclonal antibody to cross-react and bind to other major retinal Cx molecules, such as human Cx43, Cx45, Cx59, and Cx62. In addition, it further suggested a high specificity for the utilized serum to recognize Cx36 GJ sites in all mammalian models. Finally, the analysis showed that the antibody recognition site of the human Cx36 sequence is very conservative and shares high homology with Cx36 recognition sites of mammalian animal models (**Figure 2A**). This is a crucial factor when experimental animals are used as models to examine mechanisms of human vision. Besides the high similarity, Cx36 sequences of close relative animals formed separate branches, such as the carnivora branch (cat, ferret, dog) and the rodent branch (mouse, rat, hamster) (**Figure 2B**).

### Cx36 Is Expressed in All Mammalian Models

**FIGURE 3:**
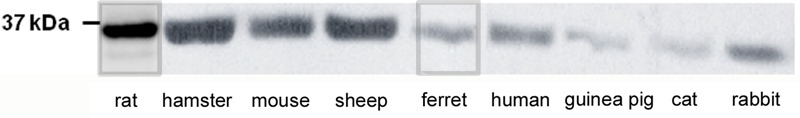
**Cx36 protein subunits are expressed in retinas of examined species.** WB analysis of Cx36 from retinas of most examined species. A single band at approximately 36 kDa was detected in rat, golden hamster, mice, sheep, ferret, human, guinea pig, cat, rabbit, (left molecular weight marker).

Next, WB was utilized to determine if significant quantity of Cx36 protein was expressed in the examined retinal samples, including human, ferret, hamster, cat, rabbit, sheep, rat, guinea pig, and mouse. WB experiments were carried out by utilizing a rabbit polyclonal antiserum raised aginst the C-terminal of the Cx36 (the monoclonal antiserum did not work in WB experiments). As expected the polyclonal antiserum showed immunoreactive bands around 36 kDa in all examined samples (**Figure 3**) suggesting that the Cx36 protein subunit is expressed in all examined samples in a detectable quantity. Although, quantitative protein measurement was not carried out, the strength of 36 kDa bands varied considerably reflecting differences in Cx36 protein quantity in mammalian retina samples.

### Three Groups of Mammalian Retinas Are Distinguished by Calretinin

To evaluate the Cx36 labels in retinae of experimental animals we utilized a CaR counterlabel. CaR that specifically marks selective neuron populations in various areas of the central nervous sytem (Eyles et al., 2002; Ulfig, 2002; Grateron et al., 2003; Džaja et al., 2014; Hladnik et al., 2014; Tóth and Maglóczky, 2014) including the vertebrate retina (Völgyi et al., 1997; Gábriel and Witkovsky, 1998; Gábriel et al., 1999; Araki and Hamassaki-Britto, 2000). CaR has also been shown to be expressed by AII cells in a number of examined mammalian species. AII cells have also been shown to maintain two populations of Cx36 GJs (Feigenspan et al., 2001; Güldenagel et al., 2001; Mills et al., 2001; Deans et al., 2002), thus the CaR counterlabel offered an opportunity to examine AII GJs in several mammals of this study. CaR is one of the main EF-hand Ca^2+^-buffer proteins, and is utilized by neurons to control the intracellular concentration levels of free Ca^2+^. Here, we used CaR to label neurons in both the GCL and INL thereby to mark the IPL boundaries and to demarcate the ONL and OPL in certain species. Once retinal layers were determined the precise location of immunolabeled Cx36 plaques were obtained.

**FIGURE 4:**
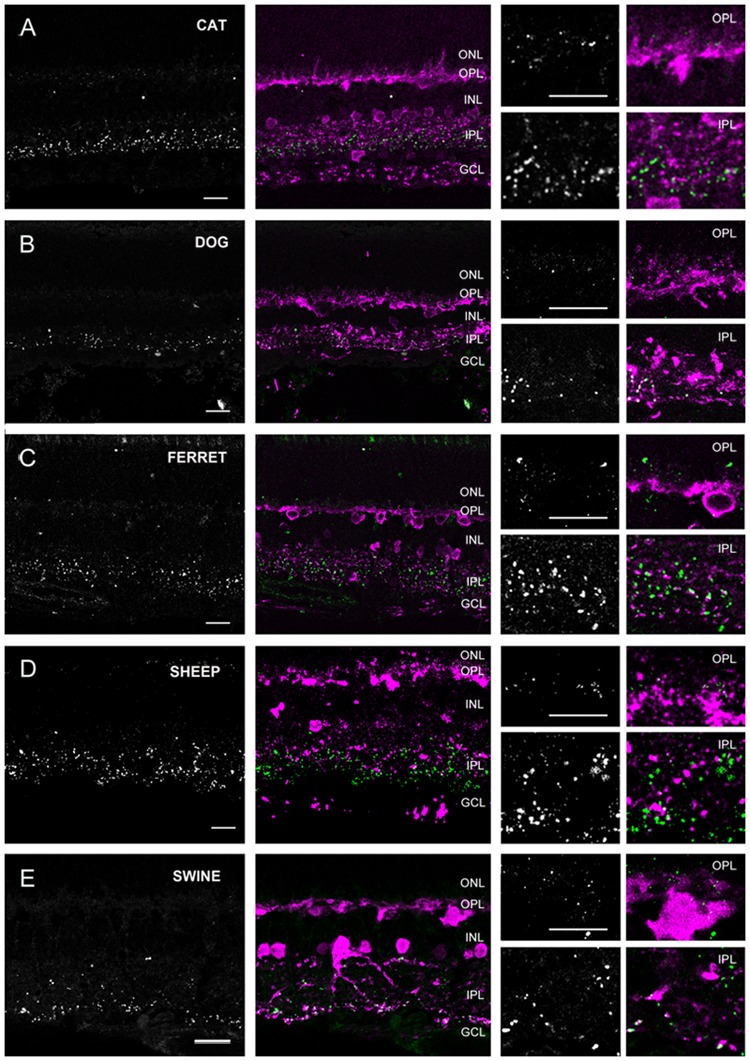
**Cx36 plaque expression in retinas of carnivore and artylodactile species. (A–E)** Retinal cross-sections display Cx36 plaques in the cat **(A)**, dog **(B)**, ferret **(C)**, sheep **(D)**, and swine **(E)** retinas in the column to the left. Column in the middle display the same images with Cx36 plaques (green) and CaR immunopositive retinal neurons (magenta). CaR expressing cells included horizontal cells in the outer retina and ACs and sporadic GCs (not shown) in the inner retina. The CaR profiles demarcated boundaries of both the OPL and IPL thus allowing for the more precise examination of Cx36 plaque distribution patterns. Insets to the right display enlarged images of Cx36 (left) and Cx36/CaR (right) labeled samples focusing on areas either in the OPL (top) or in the IPL (bottom). ONL, outer nuclear layer; OPL, outer plexiform layer; INL, inner nuclear layer; IPL, inner plexiform layer; GCL, ganglion cells layer. Scale bars: 10 μm.

**FIGURE 5:**
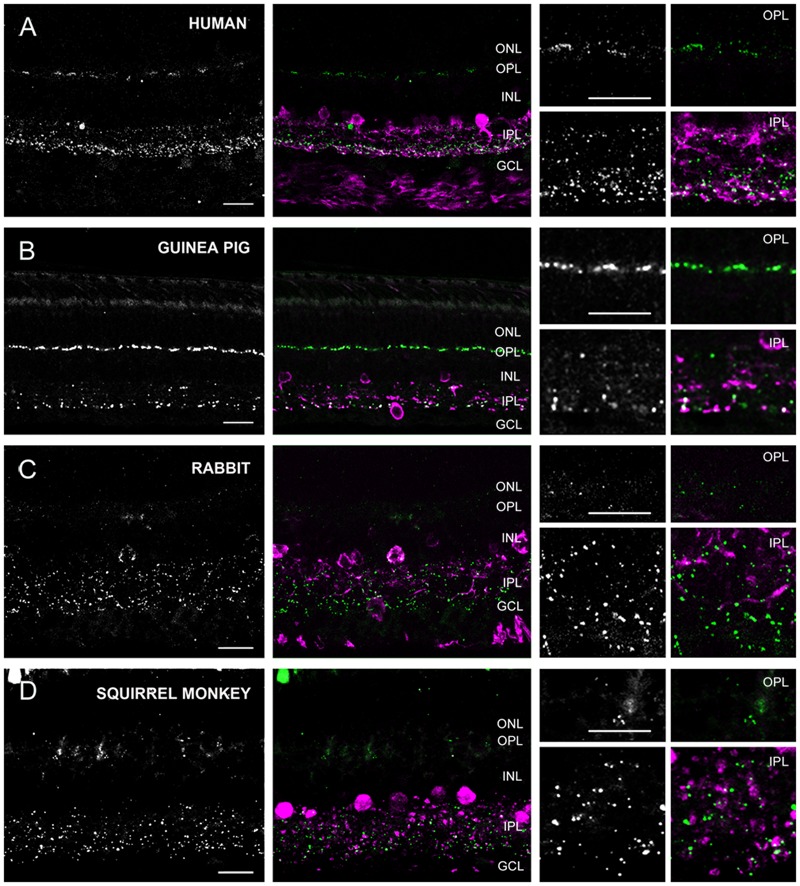
**Cx36 plaque expression in human, squirrel monkey, guinea pig and rabbit retinas. (A–D)** Retinal cross-sections display Cx36 plaques in the human **(A)**, guinea pig **(B)**, rabbit **(C)**, and squirrel monkey **(D)** retinas in the column to the left. Column in the middle display the same images with Cx36 plaques (green) and CaR immunopositive retinal neurons (magenta). CaR expressing cells included only ACs and GCs in the inner retina. These CaR+ neurons demarcated boundaries of the IPL thus allowing for the precise examination of Cx36 plaque distribution in this layer. Insets to the right display enlarged images of Cx36 (left) and Cx36/CaR (right) labeled samples focusing on areas either in the OPL (top) or in the IPL (bottom). ONL, outer nuclear layer; OPL, outer plexiform layer; INL, inner nuclear layer; IPL, inner plexiform layer; GCL, ganglion cells layer. Scale bars: 10 μm.

**FIGURE 6:**
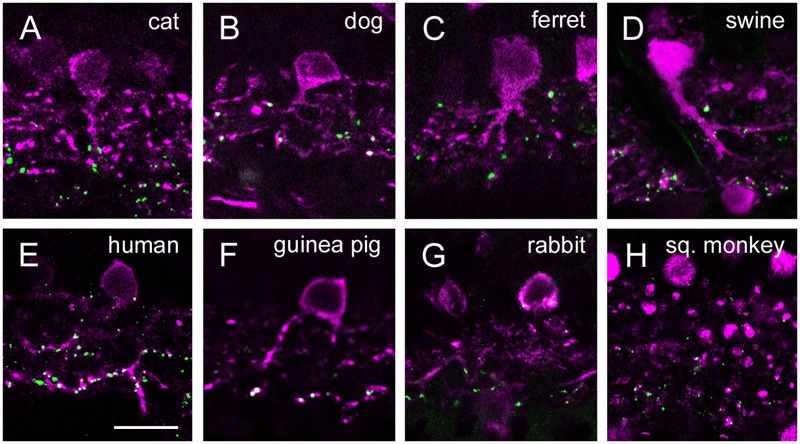
**Group 1 and 2 retinal displayed CaR stained AII-like ACs. (A–H)** CaR labeling in the inner retina of group 2 and 3 mammals displayed ACs with somata in the innermost INL, one or two thick descending primary dendritic stalks and processes that reach deep IPL strata. In most species (cat – **A**, dog – **B**, ferret – **C**, swine – **D**, human – **E** and squirrel monkey – **H**) CaR stained lobular processes could be observed in the OFF sublamina as well. These descriptions suggested that many CaR positive ACs in group 2 and 3 animals were in fact AII cells. The only exception was the sheep retina where the CaR serum labeled only dendritic processes in the IPL but somata remained unstained (not shown). Panels were generated by collapsing two **(C,E,F)** or three **(A,B,D,G,H)** 0.5 μm thin optical sections in order to show the AII-like morphology of CaR+ ACs. Scale bars: 10 μm.

**FIGURE 7:**
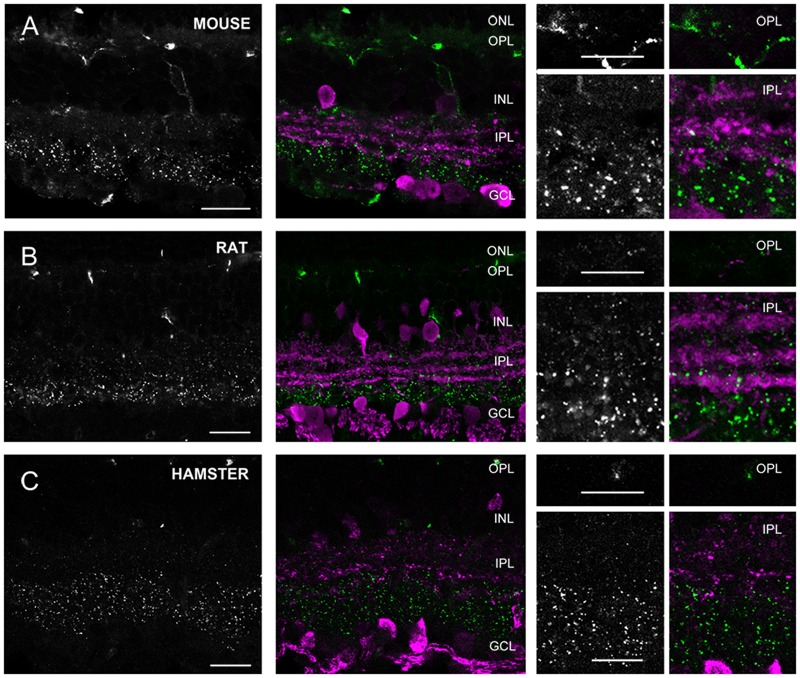
**Cx36 plaque expression in rodent retinas. (A–C)** Retinal cross-sections display Cx36 plaques in the mouse **(A)**, rat **(B)**, and golden hamster **(C)** retinas in the column to the left. Column in the middle display the same images with Cx36 plaques (green) and CaR immunopositive retinal neurons (magenta). CaR expressing cells included ACs with somata in both the INL and the GCL and narrowly stratifying CaR+ plexi in strata 2, 3, and 4. In addition CaR+ GCs were also present in the GCL. These CaR+ profiles demarcated boundaries of the IPL and also delineated the IPL strata thus allowing the precise examination of Cx36 plaque distribution. Insets to the right display enlarged images of Cx36 (left) and Cx36/CaR (right) labeled samples focusing on areas either in the OPL (top) or in the IPL (bottom). ONL, outer nuclear layer; OPL, outer plexiform layer; INL, inner nuclear layer; IPL, inner plexiform layer; GCL, ganglion cells layer. Scale bars: 10 μm.

Based on labels of CaR positive neuronal structures we distinguished three groups of species. The first group (group 1) included the cat, dog, ferret, sheep, and swine (carnivora and artiodactyla), whose retinas displayed thick, horizontally running CaR immunoreactive dendrites around the INL/OPL border and large somata in the distal INL (**Figure 4**). These features unequivocally identified these structures in all of the five species as horizontal cells. In the inner retina of group 1 animals, CaR was expressed by AC somata in both the INL and displaced to the GCL as well as GC somata in the GCL (identified by the axons in the nerve fiber layer) (**Figure 4**). In addition to these CaR positive somata, labeled processes were also discernible in the IPL. The group 2 samples included the human, the guinea pig, the squirrel monkey and the rabbit retinas (primates, lagomorpha, and some rodents) (**Figure 5**). The outer retina of these species were entirely devoid of CaR labeled structures and only the inner retina contained CaR positive ACs and GCs in the INL and the GCL, respectively. In both group 1 and 2 retinas, some of the CaR expressing ACs displayed lobular-like dendritic profiles characteristic of AII ACs (**Figure 6**). In fact, AII cells have been shown to express CaR in a number of mammalian species, including the cat, the monkey, and the rabbit (Völgyi et al., 1997; Massey and Mills, 1999; Mills and Massey, 1999; Kolb et al., 2002; Kántor et al., 2016b). Thus, we suspect that CaR positive neurons of all group 1 and 2 species that display AII-like soma- and dendrite morphology were AII cells as well. Besides AII cells, non-AII ACs, displaced ACs and GCs also showed CaR immunopositivity in these species. Group 3 animals included the mouse, the rat and the hamster (rodents), whose outer retina did not display any CaR expressing structure but the inner retina showed CaR labeled neurons in both the INL and the GCL (**Figure 7**). In contrast to group 1 and 2 retinas, however, dendritic processes of the CaR expressing inner retinal neurons showed narrow stratification in strata 2, 3, and 4 of the IPL. It has been documented that in both the mouse and the rat starburst-a and starburst-b ACs and WA-2/3 ACs can be accounted for the three CaR positive bands in the IPL (Gábriel and Witkovsky, 1998; Knop et al., 2014). To summarize these above results we conclude, that close relative species displayed similar CaR labels, for example most rodent retinas formed group 3, all carnivore species were in group 1 and the human and squirrel monkey retinas were in group 2. Thus, this comparison of the CaR distribution pattern, a commonly used neurochemical marker showed expressional similarities in the retinas of closely related species (with the sole exception of the guinea pig), whereas it exhibited major differences between distant relatives. Regardless the examined species the CaR immunolabel delineated boundaries of the IPL and it also provided a background label to demarcate the OPL in carnivores and artiodactyles and IPL strata in rodents.

### Cx36 Expression Patterns of Various Mammalian Species

The above detailed CaR expression patterns served to determine the borders of the plexiform layers and also as potential colocalization sites with Cx36 plaques. The section below will provide a species-by-species description of Cx36 patterns in retinal cross-sections for our mammalian models.

In group 1 retinas (dog, cat, ferret, sheep and swine) the OPL was clearly marked by the CaR labeled horizontal cell dendrites. We found faint and small Cx36 positive plaques in the OPL of all five species (**Figure 4**). Cx36 plaques seemed somewhat more numerous in the ferret and the cat OPL but otherwise their distributions were very similar in all five species. Although, Cx36 plaques were scattered throughout the OPL, most of them appeared to localize above the CaR positive HC dendrites in the distal OPL.

**FIGURE 8:**
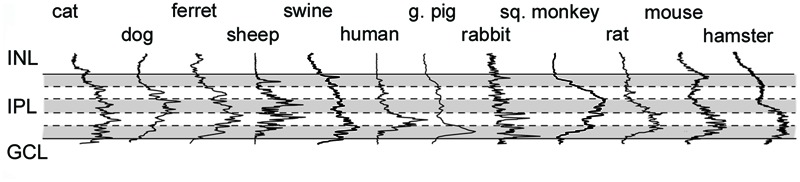
**Cx36 plaque distribution in the IPL of mammalian species.** Histogram showing results of densitometric analyses in the IPL of various mammalian species. Solid lines delineate IPL boundaries (INL/IPL on top, IPL/GCL on bottom), whereas dashed lines represent borders of IPL strata (shading helps the distinction between strata). Densitometric data were normalized to the highest measured peak, thus they only show relative Cx36 plaque density (see Materials and Methods for details).

The IPL of all five group 1 retinas showed scattered Cx36 plaques but they varied somewhat in the labeling intensity and the number of the Cx36 plaques (see below). However, regardless this variablitity the Cx36 staining was clearly stronger in the ON sublamina (**Figure 8**). We found many CaR-Cx36 double labels in the IPL, particularly in the proximal ON sublamina where AII AC transversal processes form Cx36 GJs with each other or with ON cone bipolar cell axons (**Figure 6**). Given the fact that AII ACs express CaR in group 1 animal retinas (Völgyi et al., 1997; Massey and Mills, 1999; Mills and Massey, 1999; Kolb et al., 2002; Kántor et al., 2016b) it is very likely that most of the observed double labels represent AII GJ contacts (Kántor et al., 2017).

Group 2 animals showed two subpopulations based on the Cx36 immunolabels in the OPL (**Figure 5**). Whereas the human and guinea pig retinas displayed robust Cx36 labels in the outer retina, the rabbit and the squirrel monkey retinas exhibited only sporadic and faint Cx36 puncta. Cx36 plaques in the human and guinea pig retinas were found throughout the OPL and many of them formed strip-like conglomerates that resembled subpedicle Cx36 aggregates found in some species (Feigenspan et al., 2004; O’Brien et al., 2012; Kántor et al., 2016a). Cx36 plaques scattered throughout the inner retina of all four group 2 species, however, their distributions showed some differences as well. The human and guinea pig IPLs displayed a pronounced asymmetry in the Cx36 plaque distribution with fewer plaques in the OFF sublamina and a higher number of plaques in the ON sublamina. This asymmetry in the Cx36 plaque distribution was less prominent or absent in the rabbit and the squirrel monkey retinas, respectively. To ensure that differences between the close relative human and squirrel monkey retinas did not occur due to eccentricity dependent regional differences in individual retinas we compared samples collected from peripheral and central retinal areas (all from the inferinasal quadrant) for both species. In fact, we found that regional differences did occur as central retinal samples possess more pronounced Cx36 conglomerates in the human OPL when they are compared to those of peripheral areas (**Supplementary Figure S1**). In addition, the distribution of Cx36 plaques appeared somewhat less symmetric in the peripheral than in the central squirrel monkey IPL. However, these minor regional varieties in Cx36 distribution did not alter the above detailed interspecies differences. As expected from the CaR immunolabels of these species, many Cx36 plaques in the IPL colocalized with CaR labeled cell (primarily AII AC) dendrites (**Figure 6**). Note, that only inferonasal human specimen was compared in this test, whereas neither other quadrants or the foveal region was sampled.

Group 3 animals (rat, mouse, and hamster) showed weak Cx36 plaque labels in the OPL (**Figure 7**). In contrast, all three group 3 animals displayed a large number of Cx36 plaques scattered throughout the IPL. In these animals, the CaR immunoreactive somata and IPL bands clearly marked the boundaries of IPL strata. Based on the CaR labels we found that most Cx36 immunolabeled plaques were localized in the innermost (4, 5) strata, whereas the outer IPL layers displayed immunoreactive puncta less frequently. It has been shown that CaR is majorly expressed by starburst cells of these mammalian species, and starburst cells are not GJ coupled. Thus, as expected, we did not find considerable colocalizations between the two stainings. Note, that group 3 species also displayed non-specific blood-vessel staining as well as a result of the binding of the secunder antiserum to mouse antigenes in the vessels.

### Interspecies Comparison of Cx36 Plaque Labels in the Plexiform Layers

As detailed above, based on their Cx36 labels in the OPL, the examined animals belonged to two main categories. Some animals, like cats and dogs displayed only a few solitary Cx36 plaques in the OPL (**Figures 4, 5, 7** insets). In contrast, the OPL of other species, like the human and guinea pig retinas, displayed dense conglomerates of Cx36 plaques as well as solitary Cx36 plaques. Cx36 conglomerates could also be found occasionally in the rodent OPL but solitary plaques represented the majority of the Cx36 label for these animals.

Besides the above largely qualitative description of Cx36 labels in the IPL of various species we attempted to provide a somewhat quantitative measure as well. A deeper insight into cross-species differences was obtained by the densitometric analyses of IPL Cx36 labels that were carried out in z-stacks for each species (**Figure 8**; see Materials and Methods). The normalized curves in **Figure 8** do not allow for a comparison of absolute Cx36 stainings but depict differences in relative staining intensities across the IPL strata of examined species. It is clear from this analysis that all species displayed the above mentioned ON sublayer dominance of Cx36 labels, however, this was the most pronounced in retinas of rodents as well as humans and guinea pigs. In contrast, this dominance was less obvious in the carnivora and artiodactyla retinas, where mid-IPL strata appeared to display the highest labeling intensity. In conrast to this discrepancy, the low intensity of Cx36 labels in the OFF sublamina appears to be a pan-mammalian feature.

## Discussion

### Homology of Vertebrate Cx36 Recognition Sites

Our sequence analysis revealed a great homology in Cx36 recognition sites of the presently examined animal species, thus justifying the applicability of the antiserum in a wide range of vertebrates. We also found major differences between the Cx36 recognition sites and those of similar Cx45 and Cx57 protein regions. In fact, we found that the homology of the human Cx36 subunit with paralog mammalian Cx36 sequences was significantly higher than with ortholog human Cx45, Cx57 or other connexin sequencies. The positive prediction of the sequence analysis was confirmed by the positive results of WB analyses and immunoreactions in the examined retinas. In fact, the utilized monoclonal antibody produced prominent punctate labels and little if any background staining (negative control experiments with the omission of the primary serum have been performed and no specific punctate-like staining was observed). The positive WB and IHC results here further indicate that the mammalian poly-G insert does not significantly alter the 3D conformation of the Cx36 protein and that the monoclonal antibody effectively recognized Cx36 sequences in all examined species. Despite the homology of amino acid sequences in Cx36 recognition sites across mammalian species we cannot rule out that the 3D structure of Cx36 proteines vary according to the different intracellular milieu of different retinal cells. Therefore, we cannot ensure that our antiserum in fact recognizes all Cx36 protein molecules in all examined species. This latter issue needs further experimental evidence in the future. In addition, the observed great sequential distances between Cx36, Cx45, and Cx57 fragments predicted a very low likelihood for the antibody to bind to other major retinal Cx proteins. This was also confirmed by the strong, single immunoreactive bands directly below the 37 kD marker line in the WB analysis.

### Variations in the CaR Neurochemistry of Mammalian Retinas

In order to reveal layer boundaries and mark individual IPL strata in some specimens we utilized a CaR background staining. CaR has been demonstrated to specifically label AII ACs in most mammalian species (rabbit – Völgyi et al., 1997; Massey and Mills, 1999; monkey – Mills and Massey, 1999; cat – Goebel and Pourcho, 1997; human – Kántor et al., 2016b; Lee et al., 2016), whereas starburst but not AII ACs express CaR in the rat and the mouse retina (Gábriel and Witkovsky, 1998; Haverkamp and Wässle, 2000). These observations were confirmed here showing that AII ACs in the rabbit, cat, and monkey retina express CaR, whereas AII ACs in the rat and the mouse did not. Beyond confirming previous observations, our investigation showed that AII like ACs are also CaR^+^ in guinea pig, dog, ferret, sheep, and hamster retinas. AII AC somata were located at the INL/IPL border and the terminal endings of their transversal processes form synapses with rod bipolar cells in the proximity of the IPL/GCL border. Thus, CaR^+^ AII AC labels marked both IPL bounderies in most of the examined mammalian species. Those with CaR^+^ starburst but not AII cells on the other hand displayed two populations of starburst cell somata in either the INL, or in the GCL thereby marking the INL/IPL and the IPL/GCL borders, respectively. Furthermore, the CaR stained starburst cell processes delineated strata 2 and 4 as well. In a few retinas, including the dog, the cat and the ferret, CaR was also expressed by HCs thereby precisely locating the OPL in these species. Therefore, we conclude that the CaR label is a useful tool to delineate IPL boundaries in all mammalian species and can be further utilized to label the OPL and IPL strata in certain animals.

### Various Functions Determine Cx36 Plaque Formation in the Mammalian Retina

The observed Cx36 plaques showed a rather inhomogenous distribution in the IPL of all the mammalian species. Although, Cx36 puncta can be found all over the IPL they seem considerably more prevalent in the ON sublamina in all examined species. This data thus suggests that a number of these Cx36 puncta are GJs formed between AII ACs as well as between AII cells and ON cone bipolar cells (Feigenspan et al., 2001; Güldenagel et al., 2001; Mills et al., 2001; Deans et al., 2002). This was confirmed by the observed colocalizations of CaR^+^ AII structures and Cx36 plaques in some examined species. As CaR labels starburst but not AII ACs in the retinae of the mouse and the rat (Gábriel and Witkovsky, 1998), colocalisation of Cx36 and CaR was sporadic and likely occured only by chance in these rodents. However, even in these retinas the highest density and size of Cx36 puncta occured in the proximal part of the IPL and their pattern seemed very similar to those of other mammals. Thus, this suggests that most of these Cx36 plaques in rat and mouse belong to AII AC GJs as well. An additional and indirect evidence supporting the same ‘AII GJ’ hypothesis is provided by observations in primate retinal samples (human and squirrel monkey). Primate retinas possess eccentricity dependent differences in cone/rod composition in the outer retina and thus a corresponding differences in the numbers of neurons partake in cone and rod signaling pathway. According to these differences one expects a higher number of AII ACs and AII GJs in peripheral monkey and human retinas than in the central IPL. Although, such eccentricity related difference was not obvious in the case of the human retina, the Cx36 densitometry showed a maximum in the mid-IPL in central retinas but appeared somewhat more assymetric in the periphery. This latter difference could be explained with a peripheral increase of the Cx36 density that corresponds well with the more pronounced presence of rod pathway neurons at these areas. The lack of such eccentry controlled difference in the human retina is less obvious. It is possible that the decreased number of rod pathway neurons, including AII cells, in central retinal areas are compensared by an increased number of GJ contacts each AII cells may form. In addition to the dense Cx36 immunolabels in the ON sublamina, the OFF sublamina contained numerous but small and faint Cx36 puncta in all examined mammalian species. It has peviously been reported that most retinal GCs in the mouse form GJs with GCs and/or ACs (Völgyi et al., 2009) and they preferentially contain Cx36 (Pan et al., 2010). Thus, it is very likely that most Cx36 puncta in the OFF sublamina and some of the small and relatively weakly stained Cx36 labels in the ON sublamina originate from these GC GJs.

In contrast to subtle differences in the IPL, the Cx36 expression in the OPL varied substantially accross mammalian species: from the dog, whose retina showed little if any OPL labels to the guinea pig whose OPL was richly filled with aggregated Cx36 plaques. Similar to the dog, the cat and the ferret retinas displayed only few solitary Cx36 plaques in the OPL, the mouse and rat retinas showed somewhat more, whereas the human retina (similar to the guinea pig retina) exhibited many Cx36 plaques. The observed Cx36 plaques in the OPL might be expressed by two neuron populations in mammals; cone photoreceptors that form GJs with each other and/or with rods (Schneeweis and Schnapf, 1995; Deans et al., 2002; Kántor et al., 2016a) and cone bipolar cells that maintain subpedicular bipolar-to-bipolar junctions (Feigenspan et al., 2004; Bolte et al., 2016; Kántor et al., 2016a). The above observed difference in Cx36 expression in the OPL reflects the prevalence of either of the two junction systems in the examined mammalian species. The finding that the rod dominant cat, dog and ferret retinas displayed less Cx36 labels clearly supports this hypothesis. These latter retinas possess relative few cones thus they likely have less cone-cone and cone bipolar – cone bipolar GJs. In contrast, retinas with high cone ratio, like human and guinea pig retinas have a higher number of Cx36 GJs between cones and also between cone bipolar cell dendrites.

In general, evolutionarily close relative species show similar Cx36 patterns in both the IPL and OPL: (i) Carnivores (ferret, cat, and dog) display only few Cx36 plaques in the OPL and moderate ON dominance in the IPL; (ii) rodents (rat, mouse, and hamster) have few OPL plaques and strong ON dominance in the IPL. However, there are obvious similarities among far relatives also as both the human and guinea pig OPL display lot of Cx36 plaques. Moreover, discrepancies in Cx36 expression can be found between close relatives, for example human and squirrel monkey retinas display dissimilar distribution of Cx36 plaques in both the OPL (the prevalence of subpedicular plaques) and IPL (the ON dominance of plaques). This thus, clearly shows that mechanisms other than evolutionary vicinity might determine Cx36 distribution patterns in the mammalian retina. The above presented correlation between the stronger Cx36 labels in the OPL and the high cone/rod ratio, suggests that the life-style of animals (besides evolutionary relationships) is critical to determine Cx36 expression patterns in both plexiform layers. Rodents evolutionarily are not close relatives with carnivores but they both live a nocturnal lifestyle. Thus, it is very likely that the nocturnal lifestyle and perhaps the related rod dominance of their retinas is in a direct correlation with the weak Cx36 labels in the OPL. The similarity of the Cx36 expression pattern in the human and the guinea pig OPL also supports the ‘lifestyle-hypothesis.’ Humans and guinea pigs are not close relatives but they are both diurnal. In addition to lifestyle, guinea pigs and humans share another common feature as they both are precocials whereas all other examined species are altricials. Altricials like rats and mice are born with closed eyes and their retinas almost mature when eye opening occurs. Precocials (guinea pigs and humans) on the other hand open their eyes at birth thus their immature retinas are the subject to a longer experience dependent postnatal maturation period (Racine et al., 2008). Therefore, besides the similar lifestyle, the postnatal development of the retinal tissue may also be an important factor in forming the Cx36 pattern in the mammalian OPL. Finally, it should be noted that the above listed differences in Cx36 plaque distribution patterns were observed in rather central retinal areas. Since eccentricity related variations in Cx36 plaque distributions are expected in all species it is very likely that the peripheral retina displays less prevalent species-to-species differences, whereas central specializations (fovea, visual streak, and area centralis) differ considerably.

### Applicability of Mammalian Model Animals

One goal of this study was to determine if popular animal models can be utilized in clinically oriented studies. Regarding retinal Cx36 electrical synapses, all mammalian retinas possessed some common features like the existence of plaques in both plexiform layers. Despite the similarity, subtle differences in the ON sublayer preference of Cx36 plaques and the presence of subpedicle Cx36 aggregations in the OPL was observed. The wealth of subpedicle Cx36 aggregates in the human OPL has been reported previously (Kántor et al., 2016a) and shown that these structures are GJ sites formed by bipolar cell dendrite terminal endings. GJs in subpedicle aggregates connect bipolar cell dendrites that are postsynaptic to the same cone pedicle, thus likely serve a function necessary for photopic visual signaling. Although, the presence of similar subpedicle aggregates has been reported in the macaque monkey (O’Brien et al., 2012), the retina of the new world squirrel monkey, another close relative to humans, did not show Cx36 plaque aggregations. The presence of Cx36 aggregates has been reported in the mouse retina previously (Feigenspan et al., 2004; Bolte et al., 2016), however, the density and prominance of mouse Cx36 aggregates were not comparable to those observed in the human retina. In addition to the mouse, none of the other rodents (hamster, rat) displayed human like Cx36 aggregates in the OPL. In fact, the lack of dense Cx36 aggregates was observed in all examined carnivores, artilodactyles and the lagomorphe rabbit as well. Therefore, the guinea pig was the only species in our sample that displayed human like extended aggregates of Cx36 plaques in the OPL. In this species both the apparent frequency and location of aggregates were similar to those of humans. Therefore, when cone bipolar cell signaling is examined experimentally to model human vision the optimal choice is either the macaque or the guinea pig retina. Data from any other model animal may lack much of the effects bipolar cell-to-bipolar cell GJs mediate for human vision. However, our comparison was performed only in samples collected from a spatially restricted area (central inferonasal) from all species. Since, the Cx36 plaque distribution may display region specific differences in the human retina it cannot be ruled out that the rest of the species (rodents in particular) may serve to model human vision performed by other retinal quadrants or central retinal areas. Future investigations thus have to address this issue to either further verify and generalize the above findings or pinpoint the region specific comparative aspects.

Another feature in which the examined retinas displayed subtle differences was the presence or lack of ON dominance in Cx36 plaque distribution in the IPL. In this respect, the rodent, the guinea pig and for the most part the swine retina displayed ON dominance of Cx36 plaques that appeared similar to those observed in human retinal samples. Moreover, the ON dominance is somewhat more prominent in rodents than in other species. This is probabily due to the fact that most Cx36 plaques in the IPL (particularly the large ones) serve primary rod pathway AII-AII and AII-ON cone BC GJ connections (Kovács-Öller et al., 2014), a system which is particularly well developed in rodents. Therefore, these above mentioned animals can serve as appropriate species to model the human retina when the research question involves circuits with inner retinal electrical synapses or scotopic retinal signaling.

## Author Contributions

TK-Ö: Designing and performing experiments, analyzing data, and writing paper; GD and MB: performing experiments and analyzing data; AG, JO, and MN: analyzing data; LB: collecting tissues; OK: collecting tissues and analyzing data; BV: designing experiments, analyzing data, and writing paper.

## Conflict of Interest Statement

The authors declare that the research was conducted in the absence of any commercial or financial relationships that could be construed as a potential conflict of interest.

## Footnotes

1 http://www.clcbio.com

## Supplementary Material

FIGURE S1 **Effects of eccentricity on Cx36 plaque distribution in the human and squirrel monkey retinas. (A,B)** Pair of images display the Cx36 plaque distribution in human retinal samples in peripheral **(A)** and central **(B)** retinal areas (both from the inferonasal quadrant). Little (if any) difference could be seen in Cx36 plaque distribution between peripheral **(A)** and central **(B)** human retinal areas. Plaque distribution in the IPL does not show a clear eccentricity related variation. In the OPL the subpedicle Cx36 conglomerates are readily found in central and but are less numerous in peripheral areas. **(C,D)** Pair of images display the Cx36 distribution in squirrel monkey retinal samples in peripheral **(C)** and central **(D)** areas. Central retinal areas display a somewhat more asymmetric Cx36 plaque distribution favoring the ON sublayer of the IPL. The OPL of the squirrel monkey retina is comparable in both the center and periphery in that they do not possess Cx36 plaque conglomerates. ONL, outer nuclear layer; OPL, outer plexiform layer; INL, inner nuclear layer; IPL, inner plexiform layer; GCL, ganglion cells layer. Scale bars: 10 μm.

## Abbreviations

aa: amino acid
AC: amacrine cell
CaR: calretinin
Cx: connexin
GC: ganglion cell
GCL: ganglion cell layer
GJ: gap junction
IHC: immunohistochemistry
INL: inner nuclear layer
IPL: inner plexiform layer
mCx36ConSeq: mammalian Cx36 consensus sequence
ONL: outer nuclear layer
OPL: outer plexiform layer
WB: Western blot
