# Supplementary figures and images for: Connexin36 Expression in the Mammalian Retina: A Multiple-Species Comparison
Source: Front Cell Neurosci. 2017 Mar 9;11:65. doi: 10.3389/fncel.2017.00065 (PMC5343066; doi:10.3389/fncel.2017.00065)

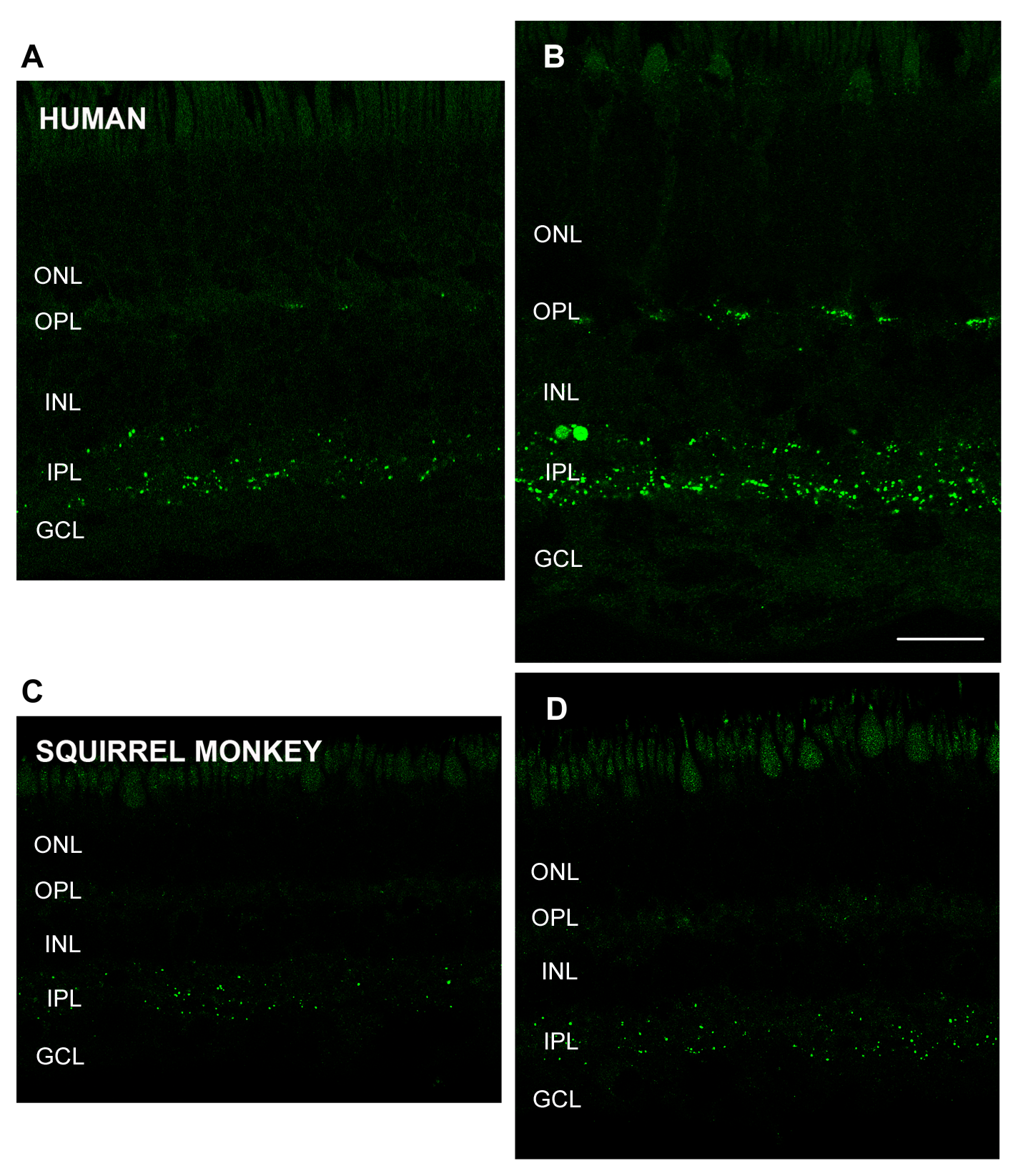

Supplement: FIGURE S1 — Effects of eccentricity on Cx36 plaque distribution in the human and squirrel monkey retinas. (A,B) Pair of images display the Cx36 plaque distribution in human retinal samples in peripheral (A) and central (B) retinal areas (both from the inferonasal quadrant). Little (if any) difference could be seen in Cx36 plaque distribution between peripheral (A) and central (B) human retinal areas. Plaque distribution in the IPL does not show a clear eccentricity related variation. In the OPL the subpedicle Cx36 conglomerates are readily found in central and but are less numerous in peripheral areas. (C,D) Pair of images display the Cx36 distribution in squirrel monkey retinal samples in peripheral (C) and central (D) areas. Central retinal areas display a somewhat more asymmetric Cx36 plaque distribution favoring the ON sublayer of the IPL. The OPL of the squirrel monkey retina is comparable in both the center and periphery in that they do not possess Cx36 plaque conglomerates. ONL, outer nuclear layer; OPL, outer plexiform layer; INL, inner nuclear layer; IPL, inner plexiform layer; GCL, ganglion cells layer. Scale bars: 10 μm. [file Image_1.TIF]
